# Selected Nutrients to Oppose Muscle Disuse Following Arthroscopic Orthopedic Surgery: A Narrative Review

**DOI:** 10.3390/nu17071273

**Published:** 2025-04-05

**Authors:** Dean M. Cordingley, Maryam Taheri, Moein Fasihiyan, Jarret M. Woodmass, Stephen M. Cornish

**Affiliations:** 1 Applied Health Sciences, University of Manitoba, Winnipeg, MB R3T 2N2, Canada; umcordid@myumanitoba.ca; 2 Pan Am Clinic Foundation, 75 Poseidon Bay, Winnipeg, MB R3M 3E4, Canada; 3 Faculty of Kinesiology and Recreation Management, University of Manitoba, Winnipeg, MB R3T 2N2, Canada; 4 Faculty of Sport Science and Health, Shahid Beheshti University, Tehran 19839 69411, Iran; 5 Department of Kinesiology and Physical Education, McGill University, Montréal, QC H2W 1S4, Canada; 6 Orthopaedic Surgery, Pan Am Clinic, 75 Poseidon Bay, Winnipeg, MB R3M 3E4, Canada; 7 Division of Orthopaedics, Department of Surgery, University of Manitoba, Winnipeg, MB R3A 1R9, Canada; 8 Centre for Aging, University of Manitoba, Winnipeg, MB R3T 2N2, Canada

**Keywords:** atrophy, creatine, omega-3, vitamin D, Glutamine, orthopedic surgery

## Abstract

**Background**: Orthopedic surgery and the corresponding events (i.e., immobilization and muscle disuse) result in a cascade of biological events to promote healing but can come with the loss of skeletal muscle mass and strength. A good nutritional status of patients is associated with positive post-surgical outcomes, with macronutrients receiving the majority of emphasis in the research literature. However, beyond the surgical literature, there are other nutrients and nutritional supplements that have been established or postulated to improve skeletal muscle mass and strength. **Objective**: The purpose of this narrative review is to provide evidence for the utility of using creatine, vitamin D, omega-3 fatty acids, glutamine, essential amino acids-branched chain amino acids (EAA-BCAA) and beta-hydroxy-beta-methylbutyrate (HMB) supplementation and the role they may play in minimizing muscle atrophy and strength loss following orthopedic surgery. The review will also highlight areas of future research to support a better understanding of the efficacy of supplementing with these substances pre- and/or post-surgery.

## 1. Introduction

Undergoing surgery triggers an inflammatory response in the immune system, leading to a catabolic state and a hypermetabolic state, necessitating an increased energy demand [1]. Elective or emergency surgery results in the loss of skeletal muscle mass and function [2,3,4] through numerous molecular mechanisms and pathways [5]. The degradation of skeletal muscle proteins poses significant health risks. To mitigate muscle atrophy during orthopedic surgery, research indicates a strong correlation between catabolic conditions in myocytes and energy intake [6]. It has been suggested that the consumption of specific macronutrients and micronutrients can effectively reduce and attenuate muscle atrophy [6].

The prolonged elevation of cortisol with surgery causes increased protein breakdown and reduced muscle protein synthesis (MPS) [7], resulting in an efflux of skeletal muscle amino acids that contribute to gluconeogenesis and immune function [8]. The increased skeletal muscle catabolism associated with surgery in combination with immobilization (or reduced mobility), occasionally associated with orthopedic surgery [9,10], could result in significant skeletal muscle atrophy [11,12]. The loss of skeletal muscle mass can occur quickly with disuse in both younger and older adults [13,14]. In young males, Kilroe et al. identified that leg immobilization with a knee brace decreases thigh muscle volume (determined by magnetic resonance imaging (MRI)) by 1.7 ± 0.3% within 2 days and continues to decrease it by ~0.8% per day for the next five days compared to the control leg [14]. Additionally, they observed a decrease in the strength of the immobilized leg for knee extension (18.7%), leg press (21%) and calf raise (8.3%) but no change in strength within the control leg was noted [14]. Dreyer et al. observed a similar response in a group of 28 older adults undergoing total knee arthroplasty where quadriceps muscle volume decreased (determined by MRI) by 14.3 ± 3.6% at 2 weeks following surgery compared to baseline [13]. Furthermore, the results indicate that individuals who experience less loss in muscle volume tend to have improved functional mobility outcomes [13]. Maintaining and/or regaining skeletal muscle mass following orthopedic surgery or muscle disuse is vital for individuals of all ages.

Nutritional supplements may serve diverse roles, with some evidence suggesting that nutrient intake can inhibit inflammatory factors, stimulate signaling pathways associated with the prevention of muscle protein breakdown (MPB), enhance MPS and improve skeletal muscle function during periods of disuse [7]. Consequently, some nutritional supplements can aid in preventing skeletal muscle atrophy and potentially facilitate recovery of patients (see Hirsch et al. for a thorough review of the role of carbohydrates and protein supplementation before and after orthopedic surgery [7]). Enhanced recovery after surgery (ERAS) to improve outcomes following orthopedic surgery has been developed and identifies the role nutrition may play in improving patient outcomes [15]. The ERAS suggest avoiding pre-operative malnutrition and ensuring sufficient carbohydrate consumption 2–3 h prior to surgery, and an early return to normal diet as tolerated [15,16]. Further evaluation is warranted considering the lack of consensus and specific recommendations regarding consuming different nutritional supplements after orthopedic surgery and during muscle disuse for muscle atrophy prevention. Creatine, vitamin D, omega-3 fatty acids, glutamine, EAA-BCAA and HMB have robust research investigating their impact on skeletal muscle health; therefore, they were identified as nutrients of interest for this narrative review. This review aims to explore the potential impact of creatine, vitamin D, omega-3 fatty acids, glutamine, EAA-BCAA and HMB supplementation in mitigating the surgical-induced cascade of intra-muscular atrophy signaling events, with the overarching goal of preserving muscle mass, optimizing inflammation and potentially enhancing functional and recovery outcomes.

## 2. Creatine

Creatine is one of the most popular and researched nutritional supplements and is postulated to confer numerous health and performance benefits. Creatine supplementation elicits physiological effects on multiple systems including skeletal muscle, bone and the brain [17]. Studies indicate that creatine supplementation may have the capacity to enhance MPS and reduce MPB [17]. Current evidence suggests that supplementing with creatine combined with resistance training can increase skeletal muscle mass [18,19,20,21] and could moderate sarcopenia and skeletal muscle atrophy [17]. Additionally, there is accumulating evidence suggesting that creatine supplementation may evoke anti-inflammatory and anti-catabolic effects [22] which would be beneficial in mitigating the cascade of events during and following surgery that leads to the loss of skeletal muscle mass.

There are few studies that have directly investigated the effects of creatine supplementation on skeletal muscle function following orthopedic surgery (Table 1). A study by Tyler et al. [23] investigated the effects of creatine supplementation (20 g/d for 7 days, followed by 5 g/d for a total of 12 weeks), initiated the day following ACL reconstruction on skeletal muscle strength and power, single-leg hop test for distance and the Knee Outcome Score (KOS). All patients (N = 60; male, n = 33 and female, n = 27; age = 30.4 ± 1.0 years) showed a loss in skeletal muscle strength and power at 6 weeks post-surgery and a subsequent improvement in both strength and power at 12 weeks and 6 months post-surgery with no difference between the creatine and placebo conditions. Additionally, there was no difference between groups in KOS or the hop test for distance at 6 months post-surgery. Taken together, these results suggest creatine supplementation does not improve functional outcomes following ACL reconstruction. Another study investigated the effects of creatine monohydrate supplementation on functional outcomes following total knee arthroplasty (TKA) [24]. Participants (Placebo, n = 19; male, n = 8; female, n = 11; age = 63.3 ± 10.2 years. Creatine, n = 18; male, n = 9; female, n = 9; age = 63.7 ± 10.0 years) were randomized to receive placebo (dextrose 7 g/d) or creatine (creatine monohydrate 10 g/d and dextrose 8 g/d for 10 days before surgery, followed by creatine monohydrate 5 g/d and dextrose 4 g/d for 30 days following surgery) supplementation. Both conditions experienced a similar decrease in body mass, fat mass and body fat percentage by 30 days post-surgery. Declines in functional outcomes also did not differ between groups, with similar decreases in ankle dorsiflexion and knee extension strength and increases in times for a 30 ft walk and 4-step climb. Histologically, both groups had similar muscle fiber areas of both type I and type II fibers 30 days post-surgery. The group that consumed creatine had a higher serum creatinine concentration 30 days post-surgery compared to placebo, but there were no differences in serum creatine kinase, gamma-glutamyltransferase, and bilirubin, as well as no differences in urine creatinine concentrations. Interestingly, there were no differences in intramuscular ATP, phosphocreatine, creatine, and total creatine concentrations 30 days post-surgery between the creatine and placebo conditions. Taken together, the two available studies indicate that creatine supplementation may not improve functional outcomes following orthopedic surgery.

Although research is limited, some evidence supports the notion that creatine supplementation during immobilization can attenuate the loss of skeletal muscle mass and strength. A small study (n = 7) by Johnston et al., that utilized a within-participant design, found that daily creatine supplementation (4 × 5 g/day) for 7 days attenuated the loss of lean body mass (creatine = +0.9% vs. placebo = −3.7%; determined by dual-energy X-ray absorptiometry (DEXA)), elbow flexor (creatine = −4.1% vs. placebo = −21.5%) and extensor (creatine = −3.8% vs. placebo = −18.0%) strength and elbow flexor endurance (creatine = −9.6% vs. placebo = −43.0%) and elbow extensor endurance (creatine = −6.5% vs. placebo = −35.0%) compared to placebo in young males following 7 days of arm immobilization [25]. These results suggest that during short periods of immobilization, creatine supplementation could help maintain skeletal muscle mass and strength which would be beneficial following surgery. However, another study illustrated that creatine supplementation (4 × 5 g/day for 7 days followed by 5 g/day for 7 days) during 2 weeks of leg immobilization did not attenuate the decrease in quadriceps muscle cross-sectional area loss or maximal knee-extension power compared to placebo in healthy young males and females [26]. Following immobilization, they had participants complete a rehabilitative knee extension program (three sessions/week for 10 weeks) while continuing to receive creatine supplementation (5 g/day) or placebo [26]. It was found that although the creatine and placebo groups experienced similar losses in skeletal muscle mass and power, the group which continued supplementing with creatine experienced faster recovery of muscle cross-sectional area and power [26]. Although it is not fully elucidated whether creatine supplementation can attenuate the loss of muscle mass during times of immobilization, it could be beneficial during rehabilitation following immobilization.

From a molecular perspective, creatine supplementation may influence several cellular processes that inhibit MPB and promote MPS. Following 10 days of creatine supplementation (20 g/day for 3 days followed by 5 g/day for 7 days) in young men, creatine supplementation increases the expression of IGF-1 mRNA and the phosphorylation of 4E-BP1 [27]. IGF-1 is a key growth factor in the regulation of anabolic and catabolic pathways [28]. IGF-1 stimulates the PI3K/Akt/mTOR and PI3K/Akt/GSK3β pathways which are important regulators of skeletal muscle hypertrophy [28]. In a human study (n = 22, age range 20–23 years) that induced skeletal muscle atrophy with 2 weeks of single-leg immobilization, creatine supplementation during immobilization (20 g/d for 2 weeks) and following immobilization during rehabilitation (15 g/d for weeks 3–6, and 5 g/d for weeks 7–12) increased muscle regulatory factor 4 (MRF4) protein content and inhibited an increase in myogenin protein content of the previously immobilized vastus lateralis muscle [26]. Thus, it appears that creatine can influence numerous myogenic mechanisms associated with anabolic and catabolic pathways.

The potential effects of creatine supplementation on surgery-induced inflammation are not clear (see Cordingley et al. (2022) for a review of the anti-inflammatory effects of creatine) [22]. In individuals with knee osteoarthritis, supplementation with creatine (20 g/d for one week followed by 5g/d for 11 weeks) does not affect C-reactive protein, tumor necrosis factor-α (TNF-α), interleukin (IL)-1β, IL-6 or s100 A8/A9 concentrations in the systemic circulation [29]. However, the combination of creatine supplementation (5 g/d) and aerobic exercise (3 d/week) for 8 weeks can reduce IL-6 and C-reactive protein in patients with heart failure [30]. The ability of creatine supplementation to modify the molecular signaling pathways associated with inflammation has not been fully elucidated, but some evidence suggests it could lead to a less pro-inflammatory environment.

Aside from supplementing with creatine post-surgery, it may also be beneficial pre-surgery. Creatine is well recognized for its ability to increase muscle mass [18,19,20,21] and could therefore be postulated as beneficial during a prehabilitation program to optimize muscle mass prior to surgery. Recently it has been identified that a subset of individuals experience inflammatory dysregulation following anterior cruciate ligament (ACL) rupture which could increase the risk of developing osteoarthritis [31]. It has also been hypothesized that inflammatory cytokines in synovial fluid at the time of ACL reconstruction may predict poor patient outcomes 2 years post-surgery [32] and are associated with poorer cartilage composition 3 years after reconstruction [33]. Previous research provides mixed results related to the anti-inflammatory effects of creatine during acute/chronic inflammatory conditions [22]; however, no studies have investigated creatine’s effects on inflammation and MPB following injury or muscle disuse conditions. Future research should explore whether creatine supplementation could protect against muscle protein degradation following orthopedic surgery or during periods of muscle disuse.

## 3. Vitamin D

Vitamin D is well recognized for its role in calcium absorption and bone mineralization [34]; however, vitamin D also supports other biological functions with many different cells expressing vitamin D metabolizing enzymes [35]. Skeletal muscle tissue expresses the vitamin D receptor [36] however the efficacy of vitamin D supplementation on skeletal muscle hypertrophy and strength is mixed [37,38,39,40,41]. Agergaard et al. [37] supplemented untrained younger (n = 20; age range = 20–30 years) and older (n = 20; age range = 60–75 years) males to consume either 46 μg (1920 IU) vitamin D_3_ and 800 mg calcium per day or 800 mg calcium alone per day for 16 weeks (latitude, 56° N) during the months of December–April. During the last 12 weeks of the supplementation protocol participants underwent a progressive resistance training protocol (3 sessions per week). Following the training protocol all groups increased quadriceps cross-sectional area (determined with MRI) with no difference in the percent change between groups for either younger (vitamin D = 11.3% vs. placebo = 7.7%) or older (vitamin D = 4.9% vs. placebo = 8.5%) participants [37]. Similar results were reported for muscle strength with all groups improving quadriceps muscle isometric strength with no difference between groups (younger, vitamin D = 6.3% vs. placebo = 8.9%; older, vitamin D = 15.0% vs. placebo = 8.4%) [37]. However, with vitamin D supplementation the authors did identify improved muscle quality (isometric strength/cross-sectional area) in the older men. In the younger men, vitamin D supplementation resulted in decreased myostatin mRNA expression and a transition in fiber type morphology where the vitamin D supplementation resulted in a greater change in the percentage of type IIa fibers compared to placebo [37].

A randomized trial in males (aged 20–30 years old; n = 20) with deficient vitamin D serum concentrations (<75 nmol/L) investigated the effects of vitamin D supplementation (4000 IU/day) on skeletal muscle functional recovery from damaging eccentric leg extension exercise [39]. Both the vitamin D and placebo treatment groups experienced decreased peak torque of knee extensor muscles following the muscle damaging protocol. However, peak knee extension torque was higher with vitamin D supplementation at 48 h and 7 days post-muscle-damaging eccentric exercise compared to the participant results from undergoing the muscle-damaging protocol prior to treatment [39]. A parallel study published in the same article utilizing isolated human myoblast cells suggests that vitamin D supplementation can stimulate cell migration, increase myotube size, and increase myonuclei accretion during muscle repair [39]. The efficacy of vitamin D supplementation in aging populations to suppress skeletal muscle atrophy and preserve muscle mass and function are mixed (for reviews, see [38,40,41]) suggesting that there may be an age-related difference in outcomes.

Immune cells express vitamin D metabolizing enzymes with vitamin D being essential for innate and adaptive immune system function with the recommendation to avoid severe vitamin D deficiency to maintain immune health and decrease the risk of autoimmune disease development [35,42]. Currently, pre-clinical models demonstrate that exposure of immune cells to vitamin D metabolites results in immune response modulation, however when evaluating in vivo human models there are not the same results [42]. Therefore, it is unclear whether vitamin D supplementation could modify the inflammatory response following injury and pre-surgical repair or following orthopedic surgery.

There are a few limited studies that support the potential use of vitamin D supplementation to help with recovery following different arthroscopic orthopedic surgeries (Table 1) [43,44,45]. Barker et al. conducted a retrospective analysis of males who underwent ACL reconstruction (n = 18), had blood samples drawn and underwent single-leg isometric strength testing ~2 weeks prior to and 3 months following ACL reconstruction. The authors found that individuals who underwent ACL reconstruction and had plasma 25-hydroxyvitamin D concentrations < 30 ng/mL had reduced strength improvements from 2 weeks pre-surgery to 3 months post-surgery compared to the individuals with plasma 25-hydroxyvitamin D concentrations ≥ 30 ng/mL [43]. Moreover, vitamin D may play a protective role in preventing injury and tissue degeneration. A retrospective cohort study found increased rates of primary ACL tears, primary reconstruction, and revision rates in patients with hypovitaminosis D [46]. Additionally, vitamin D receptor gene polymorphisms may influence the risk of developing knee osteoarthritis [47]. An individual’s milieu of inflammatory cytokines may contribute to the development and severity of knee osteoarthritis, where higher concentrations of serum TNF-α and macrophage migration inhibitory factor (MIF) are associated with increased severity [47].

Murine and in vitro models have been utilized to investigate the effects of vitamin D consumption and molecular changes associated with immobilization and skeletal muscle fiber morphology. Nakamura et al. demonstrated Vitamin D deficiency exacerbates immobilization-induced muscle atrophy in a mouse model [48]. Molecularly, they found that the expression of Atrogin-1 and MuRF1 was higher in gastrocnemius muscle of mice with low vitamin D versus standard vitamin D levels [48]. Atrogin-1 and MuRF1 are highly upregulated in catabolic conditions and are recognized to play an important regulatory role in skeletal muscle atrophy [5]. Mechanistically, in a cell culture model (C_2_C_12_ skeletal muscle cells) the addition of vitamin D (100 nM of 1.25-D3) increases muscle fiber diameter and size, as well as the expression of myogenic markers MyoD+, desmin and myogenin [49]. Additionally, vitamin D treatment resulted in the downregulation of myogenic factors IGF-1 and Mstn, while IGF-II and follistatin are upregulated [49].

The potential role of vitamin D consumption in safeguarding muscle cells and promoting muscle protein synthesis has not been fully elucidated; however, preventing the development of low vitamin D levels could be beneficial for overall health [50]. The current literature supports the need for a prospective investigation of the efficacy of vitamin D supplementation on skeletal muscle strength and cross-sectional area prior to and following orthopedic surgery.

## 4. Omega-3 Fatty Acids

The omega-3 polyunsaturated fatty acids eicosapentaenoic acid (EPA) and docosahexaenoic acid (DHA) are recognized for their many health benefits for humans [51,52]. Both EPA and DHA can be synthesized from α-linolenic acid (ALA), which is found in plant-based sources such as flaxseed and soybean [52,53], but the conversion is limited in humans and therefore consumption of foods (such as fish and shell fish) or supplements with high DHA and EPA concentrations are the most efficient methods to increase these fatty acids in the human body [54,55]. DHA and EPA are incorporated into the cellular membranes of tissues throughout the human body including skeletal muscle and mitochondria [56]. The health benefits associated with omega-3 polyunsaturated fatty acids could be through their ability to alter the activity of cellular membrane proteins [57,58] or changes in cell membrane function [59,60]. Despite the vast array of postulated health benefits of omega-3s, the typical Western diet results in a low consumption of foods containing omega-3s. Extensive research has evaluated the beneficial effects of omega-3 fatty acids on inflammation [61,62,63] and some limited research indicates omega-3 supplementation could benefit muscle hypertrophy in sedentary younger and older adults, and benefit lower body strength and function in older adults [64], but the available literature is limited and conflicting [65]. Some research supports that mechanistically omega-3 fatty acid supplementation may inhibit skeletal muscle protein degradation, stimulate muscle protein synthesis, enhance insulin sensitivity and activate skeletal muscle satellite cells [54].

An initial study by Cornish and Chilibeck [66] investigated the effects of 12 weeks of ALA supplementation (~14 g/day from flax oil) in combination with a full-body resistance training program (3 × week for 12 weeks) in 51 older (65.4 ± 0.8 years of age) males and females. The authors found that ALA supplementation in the males decreased IL-6 concentrations (62.3% decrease) and increased knee flexor muscle thickness (measured with B-mode ultrasound) to a greater extent (17.5%) compared to placebo but did not improve muscle thickness of the elbow flexors or extensors, knee extensors, muscular strength or TNF-α. In contrast, Cornish et al. [67] also completed a study to evaluate the efficacy of omega-3 supplementation (3.0 g per day) combined with a 12-week progressive resistance training protocol in older men (n = 23; age ≥ 65 years old). The results demonstrated that omega-3 supplementation had no benefit on inflammatory markers (IL-6 and TNF-α), body composition, muscle strength or skeletal muscle function compared to placebo.

Although the effects of omega-3 fatty acids on muscle hypertrophy in older adults are unclear, they may contribute to maintaining muscle mass during times of immobilization [68]. Research has suggested that omega-3 supplementation (1.86 g EPA and 1.5 g DHA per day) for 8 weeks can stimulate muscle protein synthesis in adults of various ages in the absence of resistance training [69,70]. One study found that omega-3 fatty acid supplementation (5 g/d) following 2 weeks of unilateral leg immobilization and recovery in women resulted in less muscle loss (14 vs. 8%) and higher myofibrillar protein synthesis compared to the control group [71]. Additionally, the expression of MURF-1, an apoptotic protein, was increased in the control group compared to the omega-3 group [71].

A pre-clinical murine model study by You et al. [72] investigated rats fed a fish-oil-based diet (5% cod liver oil) for 2 weeks and then subjected to 10 days of hindlimb immobilization had greater loss of muscle mass and myosin heavy chain content compared to control. Moreover, You et al. [73] found that following remobilization of the rat hindlimbs, the control group restored muscle mass and myosin-heavy chain content of the soleus muscle after 3 days, while the fish-oil-based diet resulted in delayed restoration. These findings suggest that further research is needed to elucidate the most appropriate timing of omega-3 supplementation to benefit recovery from immobilization.

One possible explanation for the varying results could be due to different doses of omega-3 fatty acids being used in studies. The current literature suggests that in older adult populations, a dose greater than 2 g/day of omega-3 fatty acids may be more beneficial to improve skeletal muscle mass than doses less than 2 g/day [74]. Further, the potential response differences between species could influence the results when various animal models are used. More research is warranted to explore the efficacy of omega-3 fatty acids and other PUFA supplementation strategies in inhibiting the breakdown of muscle proteins following orthopedic surgery and during times of disuse.

## 5. Glutamine

Glutamine is an amino acid that is found throughout the human body and is integral to immune cell function [75], where low glutamine availability contributes to impaired immune function [76]. During catabolic situations, such as following surgery, glutamine is utilized by immune cells at a similar rate to glucose [77,78]. This amino acid is crucial for various physiological processes, including protein synthesis and immune function [79], therefore possibly benefiting recovery post-orthopedic surgery.

Glutamine is a primary fuel source for rapidly dividing cells, such as immune cells and enterocytes, potentially sparing muscle tissue from catabolism [76]. Therefore, glutamine may help preserve muscle mass during the postoperative period. During severe injury and illness, low skeletal muscle glutamine concentrations were associated with poorer patient outcomes [80,81,82]. However, supplementation with glutamine can attenuate protein degradation in children with muscular dystrophy [83] indicating a possible role for supplementation in situations and diseased states where muscle mass loss may occur.

Orthopedic surgery results in an inflammatory response that glutamine may support as it can assist in promoting immune system functioning [76]. In turn, glutamine may assist in preventing post-surgical infection through the optimization of the immune system function [76]. There is some evidence to suggest that glutamine supplementation may shorten the time needed for post-surgery recovery by supporting the body’s healing processes [84].

Skeletal muscle atrophy following orthopedic surgery is common and results from a number of atrophy-inducing mechanisms [85]. Glutamine may promote skeletal muscle development and growth by increasing cell proliferation [86]. It is important to note that results are mixed, and not all studies show benefits with glutamine supplementation. The effectiveness of glutamine could depend on numerous factors such as patient age, sex, overall health, and nutritional status [87]. Additionally, mixed supplements that include glutamine as an ingredient have demonstrated benefits such as increased muscle function, enhanced protein synthesis and reduced length of hospitalization [88]. For instance, a combination of HMB, glutamine and arginine over a 4-week period prior to cardiac surgery decreased the time patients remained in the intensive care unit and hospitalized following surgery [88]. Additionally, the combination of glutamine and leucine reduces atrophy due to sepsis [89]. While, in disease-induced states of skeletal muscle atrophy (i.e., sepsis), glutamine supplementation alone may not be sufficient to mediate the loss of skeletal muscle mass [89]. However, it is unknown whether similar results would be observed with orthopedic surgery.

An in vitro study investigated the role of L-glutamine in both differentiating C_2_C_12_ myoblasts and existing myotubes and found that L-glutamine supplementation upregulates genes associated with skeletal muscle growth and survival [90]. Moreover, Myogenin, Igf-Ir and Myhc2&7 were upregulated while the expression of the Fox03 gene was restored back to baseline when skeletal muscle hypertrophy was induced [90]. L-glutamine supplementation also restored muscle cell differentiation, prevented myotube atrophy and reduced p38 MAPK [90]. The prophylactic administration of L-glutamine may be beneficial prior to undergoing orthopedic surgery. A murine study found that muscle atrophy induced by 24 h fasting in mice is reduced with L-glutamine supplementation prior to the fasting period [91]. Pre-treatment with glutamine could be another therapeutic tactic that could be valuable to accelerate/improve outcomes following orthopedic surgery but requires further research.

In summary, glutamine supplementation pre- and/or post-surgery may have potential benefits for muscle preservation, immune support and accelerated recovery. However, it is unclear whether the evidence from pre-clinical and clinical models of different disease states would translate to the orthopedic setting.

## 6. Essential Amino Acids (EAA) and Branch Chain Amino Acids (BCAA)

Essential amino acid supplements (containing all nine essential amino acids) and branched-chain amino acids (BCAA; containing leucine, isoleucine and valine) are commercially available and commonly used by the public with the hope of improving skeletal muscle mass. It is believed that the consumption of EAAs and BCAAs stimulates muscle protein synthesis, and in turn, increases skeletal muscle mass [92,93]. However, the ability of BCAAs to stimulate MPS without the inclusion of exercise in healthy younger individuals is controversial [94]. However, in older adults supplementing with BCAA, leucine may help preserve the lean mass of the lower limbs [95], help preserve insulin sensitivity and mitochondrial respiration specific pathways and resist oxidative stress associated with bed rest [96]. In patients with critical illness or muscle wasting illness, EAAs and BCAAs may be beneficial to maintaining lean body mass and total body mass and increasing MPS [97].

The evidence for the effectiveness of EAAs/BCAAs following orthopedic surgery is mixed (Table 1). The use of an EAA supplement (9 g/d; 4.5% threonine, 8.4% lysine, 6.7% isoleucine, 6.7% valine, 6.7% methionine, 2.3% tryptophan, 4.5% phenylalanine, 7.6% leucine, 3.5% histidine, 7% arginine, 12.1% glycine and 30% starch) for 1 week prior and 2 weeks following total knee arthroplasty results in increased muscle area of the rectus femoris (assessed via ultrasonography) as early as 3 and 4 weeks post-surgery compared to the placebo group [98]. Interestingly, the EAA supplementation group had greater rectus femoris muscle area and quadriceps muscle strength at 1 and 2 years post-surgery compared to placebo suggesting that optimizing nutrient content prior to and immediately following orthopedic surgery can impact long-term outcomes [98]. In patients who underwent orthopedic surgery (included in the study were patients undergoing surgery for the spine, artificial joint replacement, hip fracture and vertebral compression fracture) and rehabilitation, researchers found improved muscle quality and mass in the rectus femoris muscle with a 3 g/d EAA/BCAA supplement (1.2 g leucine with the remaining 1.8 g consisting of isoleucine, valine and lysine) compared to placebo [99]. Supplementation with a combination of 6 g EAAs (2.5 g of BCAA; 1.4 g of leucine), 5g non-EAAs (1.9 g of glutamine) and vitamin D (20 μg) consumed twice each day for 3 weeks can help preserve knee extensor and flexor strength following lumbar surgery in individuals with lumbar spinal stenosis compared to the placebo condition [100]. However, the combined supplement did not alter changes in muscle mass, gait speed or timed up-and-go performance [100].

The current literature on the efficacy of EAA/BCAA supplements to improve patient outcomes following orthopedic surgery is unclear. Randomized controlled studies are needed to establish the role that EAA/BCAAs may have in optimizing recovery from orthopedic surgery.

## 7. β-Hydroxy-β-methylbutyrate (HMB)

β-hydroxy-β-methylbutyrate (HMB) is a metabolite of leucine that may contribute to improved muscle mass and strength [101] which would be beneficial following a period of disuse and trauma following orthopedic surgery. HMB is postulated to work via numerous mechanisms to elicit positive effects on skeletal muscle. These postulated mechanisms include improving mitochondrial biogenesis, increasing cholesterol synthesis, increasing growth hormone and IGF-1, stimulating mTOR, inhibiting ubiquitin-proteasome system, decreasing apoptosis of myonuclei, increasing calcium release from the sarcoplasmic reticulum and increasing the proliferation of satellite cells [101]. These postulated mechanisms could result in preserved muscle mass following orthopedic surgery and in turn improve patient outcomes. However, the current literature is mixed regarding the ability of HMB to maintain muscle mass, strength and physical performance in older adults and those with sarcopenia (see Feng et al. [102], Mendes et al. [103] and Su et al. [104] for recent reviews).

There are currently no studies that have investigated HMB supplementation alone in the orthopedic surgery population. Nishizaki et al. (2015) investigated a mixed supplement that contained HMB as one component (2400 mg of HMB, 14,000 mg of L-glutamine, 14,000 mg of L-arginine/day) in a group of patients who underwent total knee arthroplasty [105]. The combined supplement was consumed for 5 days before and 28 days after surgery and resulted in the maintenance of leg extension strength 14 days post-operation compared to the placebo group which saw a decrease in strength (Table 1). However, due to the composition of the supplement, it is not possible to determine the relative contribution of HMB to the observed outcome.

Some studies have investigated HMB supplementation within other diseases and clinical populations who experience skeletal muscle atrophy. One study investigated 3 g/day HMB supplementation for 28 days in multi-trauma patients admitted to the intensive care unit [106]. It was found that there was no difference in quadriceps muscle layer thickness between the placebo and treatment groups, but the mean difference was slightly greater for the treatment condition at 28 days of treatment (mean difference = 0.26 cm; 95% CI, −0.13–0.64) [106]. Meza-Valderrama et al. (2024) supplemented older adults (>60 years of age), who were diagnosed with sarcopenia and discharged from a geriatric rehabilitation program, with 3 g/day HMB or placebo for 12 weeks while they underwent a resistance exercise-focused training program [107]. There was no difference between the control and HMB group for body composition, gait speed, functional status, or quality of life. However, the group who received HMB had better balance, chair stand test and total test results on the short physical performance battery compared to the placebo group [107]. These results suggest that although adaptations to muscle mass and strength may not be present, some measures of performance may be improved with HMB supplementation. Standley et al. (2017) investigated whether HMB supplementation (3 g/day) can change skeletal muscle atrophy, mitochondrial dynamics and content and intramyocellular lipids in older adults (>60 years of age) following 10 days of bed rest and 8 weeks of resistance training [108]. There was no difference in skeletal muscle fiber cross-sectional area between the HMB and placebo groups; however, following the 8-week resistance training program, HMB stimulated greater mitochondrial oxidative phosphorylation content and dynamics, and triacylglycerol compared to the placebo condition [108]. Interestingly, the same research group has shown that older adults (>60 years of age) supplemented with HMB (3 g/day initiated 5 days prior to bed rest) have improved maintenance of lean body mass at the end of 10-day bed rest compared to placebo [109]. There was, however, no difference between the HMB and placebo conditions for protein synthesis, muscular strength, or functionality following bed rest [109].

The current mechanistic evidence suggesting that HMB supplementation could reduce MPB to preserve skeletal muscle mass and strength is intriguing for the orthopedic patient population. Therefore, future investigations utilizing HMB treatment either prior to and/or following orthopedic surgery would be beneficial.

## 8. Conclusions and Future Directions

The current evidence supports the importance of adequate protein and carbohydrates in addressing the surgical cascade and supporting the maintenance of muscle mass, strength, and function [7]. Nonetheless, research investigating the effects of other nutrients around the time of arthroscopic orthopedic surgery is limited. Available data indicate that creatine, vitamin D, omega-3s and different amino acid supplements may preserve skeletal muscle protein during a muscle disuse period. High-quality randomized trials are required to inform the optimal timing, dosage and conditions (i.e., with or without limb immobilization; younger vs. older patients; type of surgical procedure) required for clinical use. An important consideration is that reviewed supplements all have either confirmed or postulated anti-atrophic effects.

In general, the results of past research have shown that supplements derived from amino acids and proteins inhibit the process of proteolysis, and vitamin supplements and derivatives of fatty acids are related to the inhibition of inflammatory factors. In addition, it seems consumption of compound supplements may have synergistic effects. Additionally, many studies in other populations show additional benefits when nutrient supplements are utilized in combination with physical exercise; therefore, the inclusion of rehabilitation exercises is important for the maintenance/recovery of muscle mass after orthopedic surgery.

**Table 1:** Studies investigating specific nutrient effects after orthopedic surgery.

Reference	Design	Nutrient of Interest	Dose	Surgical Procedure	Participants	Outcomes
Tyler et al. [23]	RCT	Creatine	20 g/d for 7 days, followed by 5 g/d for a total of 12 weeks starting the day after surgery	ACL Reconstruction	N = 60; male, n = 33 and female, n = 27; age = 30.4 ± 1.0 years	No difference in muscle strength (knee extension, knee flexion, hip flexion, hip abduction and hip adduction) or power (knee extension and knee flexion) at 12 weeks post-surgery compared to placebo. No difference in the single-leg hop test and the Knee Outcome Score at 6 months post-surgery compared to the placebo.
Roy et al. [24]	RCT	Creatine	Pre-surgery (10 g/d for 10 days), post-surgery (5 g/d for 30 days)	TKA	N = 37; placebo, n = 19; male, n = 8; female, n = 11; age = 63.3 ± 10.2 years. Creatine, n = 18; male, n = 9; female, n = 9; age = 63.7 ± 10.0 years	No difference in Δ body mass, Δ FM and Δ body fat compared to placebo. No difference in grip strength, dorsiflexion strength, knee extension strength, 30-ft walk and 4-step climb 30 days post-surgery compared to the placebo. No difference in muscle fiber area 30 days post-surgery compared to the placebo. Increased serum creatinine concentration 30 days post-surgery compared to the placebo. No difference in creatine kinase, gamma-glutamyltransferase and bilirubin. No difference in urine creatinine 30 days post-surgery compared to the placebo. No difference in intramuscular ATP, phosphocreatine, creatine and total creatine concentrations at 30 days post-surgery compared to the placebo.
Barker et al.	Secondary analysis of an RCT	Vitamin D	N/A. Investigated outcomes in individuals with high (≥30 ng/mL) and low (<30 ng/mL) vitamin D concentrations	ACL Reconstruction	N = 18, all male; age = 32 ± 2 years.	Three months post-surgery plasma interferon-gamma concentrations are higher in individuals with higher vitamin D concentrations. Isometric peak force of the injured limb increased from 2 weeks pre-surgery to 3 months post-surgery in the high vitamin D group but not the low vitamin D group.
Albright et al. [46]	Retrospective cohort study	Vitamin D	N/A. Identification of individuals diagnosed with vitamin D deficiency from the M151Ortho dataset.	ACL	N = 328,011, Female = 65.8%; age = 41.9 ± 12.6 years	Individuals with vitamin D deficiency are at an increased risk of ACL tears and revision ACL reconstruction compared to individuals who are not deficient in vitamin D.
Ueyama et al. [98]	RCT	EAA	9 g/d of EAA (threonine 4.5%, lysine 8.4%, isoleucine 6.7%, valine 6.7%, methionine 6.7%, tryptophan 2.3%, phenylalanine 4.5%, leucine 7.6%, histidine 3.5%, arginine 7%, glycine 12.1% or starch 30%) or placebo (lactose).	TKA	EAA group (n = 26, female, n = 19; age = 76.4 ± 8.3 years) and placebo group (n = 26, female, n = 23; age = 75.2 ± 5.5 years)	At 2 years post-surgery, there were no differences between groups for absolute values of rectus femoris muscle area or diameter, quadriceps muscle strength, knee pain, 6 m walk, knee range of motion or activities of daily living. Relative to baseline values, EAA supplementation resulted in greater rectus femoris muscle area and diameter 1-year and 2-years post-surgery, and greater quadriceps muscle strength 2-years post-surgery.
Ikeda et al. [99]	RCT cross-over	BCAA	BCAA 3 g/d (leucine 40%, isoleucine, valine and lysine 60%) or 1.2 g/d placebo (starch) for 1 month with a 1-week wash-out period before crossover	Orthopedic surgery for a fracture or trauma, vertebral compression fracture spinal surgery and joint replacement	N = 30; early BCAA group, n = 18; female, n = 14; age = 75.7 ± 17.8 years. Late BCAA group, n = 12; female, n = 9; age = 76.1 ± 15.0 years.	Following supplementation with BCAA for 1 month in combination with exercise therapy, participants had greater echo intensity of the rectus femoris muscle compared to their placebo condition. There was no difference between conditions for muscle mass, knee extension or grip strength, timed up-and-go test or functional independence measure.
Minetama et al. [100]	RCT	EAA/BCAA and Vitamin D	EAA/BCAA and Vitamin D (EAA 12 g/d (BCAA 5 g and leucine 2.8 g) non-EAA 10 g/d (glutamine 3.8 g) and vitamin D 40 μg/d) or placebo (vitamin D 2.5 μg/d) for 3 weeks starting the day after surgery	Lumbar surgery for lumbar stenosis	EAA/BCAA group, n = 40; female, n = 20; age = 69.8 ± 8.9 years. Placebo group, n = 40; female, n = 20; age = 71.1 ± 7.5 years.	The group that consumed EAA/BCAA and vitamin D had less muscle strength loss 2 weeks post-surgery and promoted improved strength of the knee extensor and flexor muscles by 12 weeks post-surgery, but did not influence physical function, symptom severity muscle mass loss timed-up and-go, maximal gait speed or grip strength.
Nishizaki et al. [105]	RCT	HMB, L-Arginine and L-Glutamine	2400 mg of HMB, 14,000 mg of L-glutamine, 14,000 mg of L-arginine/day consumed for 5 days before and 28 days after surgery	TKA	N = 23; female, n = 12; male, n = 11; age = 70.5 ± 5.4 years	Leg extension strength was maintained 14 days post-surgery with the supplement. Placebo condition had a decrease in leg extension strength.

RCT: randomized controlled trial; ACL: anterior cruciate ligament; TKA: total knee arthroplasty; EAA: essential amino acid; BCAA: branched-chain amino acid; FM: fat mass.

In conclusion, the findings of this narrative review suggest that nutritional supplementation interventions may serve as a viable strategy to mitigate or inhibit muscle atrophy during periods of skeletal muscle disuse and should be considered by surgeons or other health care providers when educating patients. It is recommended that future prospective randomized controlled studies examine the effects of consuming these types of supplements in preserving skeletal muscle mass and function before and after arthroscopic orthopedic surgical procedures. Additionally, studies should investigate different orthopedic surgical procedures (i.e., anterior cruciate ligament reconstruction, rotator cuff repair, joint replacement, etc.) to account for differences in invasiveness and duration of disuse (or limited use) between procedures as there is currently minimal research addressing these areas.
